# Effectiveness of the polysaccharide pneumococcal vaccine among HIV-infected persons in Brazil: a case control study

**DOI:** 10.1186/1471-2334-7-119

**Published:** 2007-10-23

**Authors:** Maria Amelia SM Veras, Wayne TA Enanoria, Euclides A Castilho, Arthur L Reingold

**Affiliations:** 1 Division of Epidemiology, School of Public Health, University of California, Berkeley, USA; 2 Preventive Medicine Department, School of Medicine, University of São Paulo, Brazil; 3 Social Medicine Department, School of Medical Sciences of Santa Casa of São Paulo, Brazil; 4 Emilio Ribas Infectious Diseases Institute, São Paulo State Health Department, São Paulo, Brazil

## Abstract

**Background:**

Polysaccharide pneumococcal vaccine is recommended for use in HIV-infected adults in Brazil but there is uncertainty about its effectiveness in this patient population.

The main objective of this study was to assess the effectiveness of the 23-valent polysaccharide pneumococcal vaccine against invasive pneumococcal infection among HIV-infected adult patients in São Paulo, Brazil.

**Methods:**

A case-control study of 79 cases and 242 controls matched on CD4+ cell count and health care setting was conducted. Among HIV-infected adults in São Paulo, Brazil, with and without S. pneumoniae recovered from a normally sterile site; prior receipt of 23 valent polysaccharide pneumococcal vaccine was determined by review of medical records and patient interview.

**Results:**

After adjustment for confounding factors, the point estimate for the effectiveness of 23 valent polysaccharide vaccine among HIV-infected adults against all invasive pneumococcal infection was 18% (95% CI: <0 to 62%).

**Conclusion:**

We were unable to demonstrate a statistically significant protective effect of 23 valent polysaccharide against invasive pneumococcal infection vaccine among HIV-infected adults in Brazil.

While the vaccine is relatively inexpensive and safe, its effectiveness among HIV-infected adults in Brazil is uncertain.

## Background

It is well-established that HIV-infected individuals are at increased risk of bacterial pneumonia in general and of pneumonia due to S. pneumoniae in particular [1,2]. The incidence of invasive pneumococcal disease (i.e. documented S. pneumoniae infection of a normally sterile site, such as blood or cerebrospinal fluid) among HIV-infected patients not receiving anti-retroviral therapy or antibiotic prophylaxis is estimated to be at least 100 times that in the general population [3-6]. In developing countries, S. pneumoniae is one of the most common opportunistic infections and is a leading cause of morbidity and mortality among HIV-infected adults and children.

Purified pneumococcal polysaccharide vaccines have been developed and licensed for use in various countries over the past 25 years. The currently available pneumococcal polysaccharide vaccine contains the 23 serotypes of S. pneumoniae that collectively account for approximately 90% of invasive pneumococcal disease in adults in the United States. This vaccine, which was licensed in the U.S. in 1983, is recommended for use in HIV-infected individuals over the age of two years by the Advisory Committee on Immunization Practices of the U.S. Public Health Service [7]. Similar recommendations for its use in HIV-infected individuals exist in Europe, Australia, and Brazil [8].

Observational studies have produced estimates of the effectiveness of the 23 valent pneumococcal polysaccharide vaccine against invasive pneumococcal infection in the range of 30% to 80% among adults with normal immune function [9-12]. However, among severely immunosuppressed adults, the polysaccharide vaccine has little or no protective effect [10]. Studies of the vaccine's effectiveness among HIV-infected adults have produced widely disparate results, perhaps in part due to varying levels of immunosuppression among the study populations [13-21]. The only randomized controlled trial of the vaccine among HIV-infected adults, conducted in Uganda, showed no efficacy against invasive pneumococcal disease and an inexplicable increase in the risk of all cause pneumonia among vaccine recipients [22,23]. Given the disparate results of earlier studies of the effectiveness of the pneumococcal polysaccharide vaccine among HIV-infected persons, we were interested in assessing its effectiveness among this patient population in São Paulo, Brazil. Because it would be considered unethical to conduct a randomized placebo-controlled trial of a vaccine that is now considered standard of care, we conducted an observational study, using a case-control approach [24].

## Methods

### Participating Institutions and Case Findings

Between November, 2000 and August, 2004, we identified invasive S. pneumoniae infections among HIV-infected adults receiving clinical care at the seven largest public health referral services for HIV/AIDS in São Paulo City and Campinas (60 km from São Paulo City). At these institutions (Emilio Ribas Institute, HIV/AIDS Referral and Training Centre (CRT), Clinical Hospital of University of São Paulo Medical School (HC FMUSP), Clinical Hospital of University of Campinas (HC UNICAMP), Hospital of São Paulo State Employee (HSPE) and Casa da AIDS of the University of São Paulo, all patients with documented HIV-infection receive care from special teams of health professionals in inpatient and outpatient clinics specifically designated for them. In all of these institutions, patients have access to comprehensive free care, including anti-retroviral medications and vaccines. The facilities include microbiology laboratories employing routine diagnostic methods. All of the participating institutions have vaccine services, which are part of a special vaccine referral network through which products not available at the routine immunization services (such as the pneumococcal polysaccharide) are provided. These vaccine services maintain separate records in which the name, age, address, vaccine, number of doses, and information on underlying health conditions, including HIV-infection status, are included.

At each participating institution, an up-to-date database of HIV-infected patients being followed is maintained. This database includes patients' names, date(s) of HIV testing; dates and results of CD4 lymphocyte cell counts; demographic data; and other clinical information.

Over the course of the three year study period, all HIV-infected patients being followed at one of the participating institutions who developed a laboratory-confirmed invasive pneumococcal infection (i.e. recovery of S. pneumoniae from a normally sterile body site) were identified by the clinical microbiology laboratory. In all instances, the decision to test a patient for the presence of S. pneumoniae or other bacterial agents (i.e. to order a culture) was made by the patient's physician(s) during the clinical management of the patient.

### Study Subjects

#### Cases

A case was defined as any HIV-infected individual over 18 years of age with invasive pneumococcal disease, defined as recovery of S. pneumoniae from a normally sterile site (e.g. from blood, pleural fluid, spinal fluid, or pericardial fluid). Cases had to have had their HIV infection status confirmed prior to the invasive pneumococcal infection.

#### Controls

Controls were defined as HIV-infected individuals over 18 years of age, without a history of documented or suspected invasive pneumococcal disease, receiving medical care at the same institution as the case and matched to the case by level of CD4 lymphocyte cell count (<200 cells/mm^3^; 200 ≤ 499 cells/mm^3 ^and ≥ 500 cells/mm^3^) measured during the same month (± 30 days).

### Subject Enrollment

For each potentially eligible case, the patient's clinical provider was asked to seek the patient's permission to be approached by the research staff. If the patient agreed, he/she received a detailed explanation about the study and signed a written informed consent prior to enrollment in the study. If the patient had died or was not mentally competent to give informed consent, consent was requested from the patient's next of kin or the individual who had legal authority to consent on behalf of the patient.

For each case enrolled in the study, we attempted to identify four possible control subjects, based on a list of CD4 cell counts generated by the laboratory at each institution. From the list of potential controls, four were randomly selected; enrollment procedures were identical to those used for cases. Potential control subjects who agreed to be contacted were called and arrangements were made to meet them at their next regularly scheduled outpatient visit, at which time written informed consent was obtained.

The study protocol was approved by the Institutional Review Boards at the University of California, Berkeley and at the institutions where cases and controls were selected, as well as the Brazilian National Ethical Committee.

### Data Collection

We interviewed patients (or next of kin) and abstracted data from medical records, laboratory records, and vaccination service files. The interview was administered by a trained interviewer using a structured questionnaire with questions concerning level of education; measures of socio-economic status and household crowding; previous hospitalization(s) with pneumonia, sexual behavior and injection drug use; contact with children; and history of vaccination. Medical and laboratory records were reviewed and information concerning clinical, demographic and behavioral characteristics, such as mode of HIV transmission, co-morbid conditions, markers of disease progression, use of antiretroviral therapy and receipt of antimicrobial prophylaxis and vaccines was collected. Vaccine exposure was assessed by patient interview, review of medical records, and review of records from the adult vaccination service, which records all vaccinations given to adults.

### Laboratory Methods

The Instituto Adolfo Lutz, the public health referral laboratory of the São Paulo State Health Department, confirmed the identification of S. pneumoniae isolates and performed the serotyping. The identification and serotyping of S. pneumoniae was done using the Quellung reaction, based on serotype-specific anti-capsular polysaccharide antisera produced by the Statens Serum Institut, Copenhagen.

### Statistical Analysis

To examine the association between prior receipt of pneumococcal polysaccharide vaccine and the development of invasive pneumococcal disease, matched bivariate analyses were performed using conditional logistic regression. Matched odds ratios (ORs) and 95% confidence intervals (CIs) were calculated. Risk factors and potential confounders for invasive pneumococcal disease were determined *a priori*. Potential confounders examined included demographic variables, educational level, smoking and alcohol use, injection drug use (IDU), report of a previous hospitalization with pneumonia; close contact with a child in the prior four weeks; history of pulmonary tuberculosis and antiretroviral use at the last visit. Vaccine efficacy was calculated as 1-OR [24]. All statistical analyses were performed using Stata version 8 (Stata Corp, College Station, Texas).

## Results

During the study interval, cultures from sterile sites positive for S. pneumoniae were identified from 99 HIV-infected patients 18 years old or greater. We were able to include 79 (80%) of these patients in our study. Of the 20 patients not included in the study, two had HIV-infection identified concurrently with the diagnosis of invasive pneumococcal disease and thus had not previously had their CD4+ T cell counts measured and were not eligible for enrollment; two were homeless and were lost to follow-up; and in one case the family refused to give consent. The other 15 patients received care only in the emergency room; were not registered at the institutions; and did not return for follow-up care. No age or gender differences were found between the 79 cases included and the 20 not included in the present study (data not shown). An average of three matched controls (range 1–4) could be identified and enrolled for each case. In total there were 242 controls. Among the 79 cases, bacteremia was the most common clinical manifestation (n = 69, 87%), followed by meningitis (n = 7, 9%). Of the 79 cases, 15 died, 13 of them with bacteremia and 2 due to meningitis.

As expected, the cases and controls had similar CD4 cell counts (median = 160 cells/ml^3 ^for cases, 174 cells/ml^3 ^controls). Age, gender, and race did not differ among cases and controls (Table 1). Among the socio-economic and demographic risk factors examined, invasive pneumococcal infection was associated with having had less education and living in sub-standard housing (i.e. being homeless or living in a "slum"). Other risk factors for invasive pneumococcal infection included alcohol consumption, injection drug use, close contact with children under the age of 10 years in the previous four weeks, and a history of previous hospitalization for pneumonia. Use of anti-retroviral medication and receipt of pneumococcal polysaccharide vaccine were each associated with a decreased risk of invasive pneumococcal infection on bivariate analysis. Among vaccinated individuals, the duration time between vaccination and infection (cases) or enrolment (controls) ranged from 35 to 988 days. The vaccine effectiveness was 63% (95% CI: 28%-81%).

**Table 1:** Characteristics of Study Participants

**Characteristic**	Cases (79)* n %	Controls (242)* N %	**OR Crude**	**95% C.I.**
Age (yrs)				
≤ 38	42 (53.16)	131 (54.13)	1.0	0.64 – 1.76
≥ 39	37 (46.84)	111 (45.87)	1.06	
Gender				
Female	30 (37.97)	88 (36.36)	1.0	0.58 – 1.60
Male	49 (62.03)	154 (63.64)	0.96	
Education				
University	5 (6.94)	42 (17.57)	1.0	
High	18 (25.00)	81 (33.89)	1.89	0.64 – 5.55
Middle	22 (30.56)	61 (25.52)	3.07	1.05 -9.00
Elementary	27 (37.50)	54 (22.94)	5.18	1.72 – 15.57
Illiterate	0 (0.00)	1 (0.42)	--	--
Race/Ethnicity				
White	48 (61.54)	156 (67.24)	1.0	0.73 – 2.16
"Non-White"	30 (38.46)	76 (32.76)	1.25	
Housing				
Standard	61 (81.33)	228 (94.61)	1.0	1.78 – 9.94
Substandard	14 (18.67)	13 (5.39)'	4.20	
Ever smoked				
No	22 (31.88)	105 (42.98)	1.0	0.85 -2.72
Yes	47 (68.12)	137 (57.02)	1.52	
Alcohol consumption				
No	63 (79.75)	221 (91.32)	1.0	1.42 – 5.86
Yes	16 (20.25)	21 (8.68)	2.88	
Injection drug user				
No	50 (69.44)	208 (87.03)	1.0	1.48 – 5.28
Yes	22 (30.56)	31 (12.97)	2.79	
Close contact w/child				
No	38 (52.78)	176 (87.03)	1.0	1.53 – 5.14
Yes	34 (30.56)	66 (12.97)	2.81	
Prev. hosp w/pneumonia				
No	41 (56.94)	191 (78.93)	1.0	1.58 – 4.98
Yes	31 (43.06)	51 (21.07)	2.80	
ARV** at the last visit				
No	28 (35.44)	34 (14.05)	1.0	0.14 – 0.49
Yes	51 (64.56)	208 (85.95)	0.26	
Received pneumo vaccine				
No	65 (82.28)	157 (64.88)	1.0	0.19 – 0.72
Yes	14 (17.72)	85 (31.12)	0.37'	

*Differences in numbers are due to missing data for specific variables

**ARV: Antiretroviral therapy

In the multivariate analysis, close contact with a child, injection drug use, and previous hospitalization for pneumonia, were still associated with an increased risk of invasive pneumococcal infection (adjusted odds ratios of 3.22 (95% CI 1.60-6.48, 2.43 [95% CI: 1.13-5.23], and 2.23 [95% CI: 1.16-4.30], respectively). Ever having pulmonary tuberculosis was no longer associated with an increased risk for invasive pneumococcal infection (Table 2). Being on anti-retroviral therapy at the time of the last outpatient visit was consistently associated with a decreased risk of invasive pneumococcal infection (OR = 0.27; 95% CI: 0.12 – 0.59). After controlling for the other variables included in the conditional logistic regression model, receipt of pneumococcal polysaccharide vaccine was no longer associated with a reduced odds of invasive pneumococcal disease. (OR = 0.82, 95% CI: 0.38 – 1.77). It is worthwhile to mention that the inclusion of demographic and socio-economic variables in the model did not change the measure of association (OR = 0.82; 95% CI: 0.33 – 2.04) (data not shown).

**Table 2:** Selected risk factors for invasive pneumococcal disease in a multivariate (conditional logistic) regression model

Characteristic	Crude OR (95% C.I.)	Adjusted OR (95% C.I.)
Pulmonary Tb ever	2.54 (1.43 – 4.50)	1.95 (0.95 – 4.03)
Close contact with child	2.81 (1.53 – 5.14)	3.22 (1.60 – 6.48)
Injection drug use	2.79 (1.48 – 5.28)	2.43 (1.13 – 5.23)
Previous hospitalization with pneumonia	2.80 (1.58 – 4.98)	2.23 (1.16 – 4.30)
Antiretroviral therapy at the last visit	0.26 (0.14 – 0.49)	0.27 (0.12 – 0.59)
Had received pneumococcal polysaccharide vaccine	0.37 (0.19 – 0.72)	0.82 (0.38 – 1.77)

Of the 79 patients included in the case-control study, S. pneumoniae isolates from 47 were available for serotyping. Of these 47 isolates, 40 (85%) were of serotypes included in the 23 valent pneumococcal polysaccharides vaccine. (Table 3). All 11 isolates from previously vaccinated cases were of serotypes included in the vaccine, as were 29 (80%) of the 36 isolates from unvaccinated cases. A sub-analysis limited to cases caused by serotypes included in the vaccine showed no evidence of a protective effect (data not shown).

**Table 3:** Pneumococcal serotype distribution according to vaccination status

Serotype	Vaccination status	
Vaccine-serotypes	Non-vaccinated	Vaccinated

1	2	2
3	2	1
5	0	1
6B	5	2
7F	2	0
9N	0	1
9V	7	1
10A	2	1
11A	2	0
14	2	1
17F	1	0
19A	1	0
19F	1	1
23F	2	0
Total vaccine serotypes	29	11
Non-vaccine serotypes		
6A	1	0
10F	1	0
13	1	0
18A	1	0
23B	1	0
Total non-vaccine serotypes	7	0
Non-typed		
G	1	0
I	1	0
Total	36	11

## Discussion

The effectiveness of pneumococcal purified polysaccharide vaccines in immunosuppressed individuals has been the subject of controversy for many years. One review and meta-analysis of the results of randomized controlled trials of pneumococcal polysaccharide vaccine effectiveness reported that while the vaccine was approximately 84% effective against pneumococcal pneumonia and 82% effective against pneumococcal bacteremia among immunocompetent individuals, it did not reduce the risk of pneumococcal pneumonia, pneumococcal bacteremia, or pneumonia deaths among those likely to have an impaired immune system [25]. Furthermore, the only randomized controlled trial of the vaccine among HIV-infected individuals (who were not receiving anti-retroviral therapy) not only failed to demonstrate any efficacy of the vaccine, but unexpectedly found an increased risk of all-cause pneumonia in the vaccinated group compared to those given placebo [22]. Longer term follow up of study participants confirmed the excess of all-cause pneumonias in the vaccinated group, but nevertheless found suggestive evidence of a reduced risk of all cause mortality among vaccine recipients [23]. Recent findings of a retrospective cohort study carried out between 1998 and 2001, in Washington State, USA, and of a case-control study conducted in Catalonia, Spain between 2001–02 supported prior studies demonstrating the effectiveness of the vaccine for the prevention of bacteremia among the elderly [11,12]. A recently published observational cohort study of HIV-infected individuals in Baltimore, Maryland did not find an association between receipt of pneumococcal vaccination and a reduced risk of invasive pneumococcal disease [21].

In general, randomized, placebo-controlled trials of pneumococcal polysaccharide vaccines among HIV-infected individuals have been judged to be not feasible in many countries for a variety of ethical and scientific reasons. As a result, most evaluations of the effectiveness of the vaccine among HIV-infected individuals have been conducted using observational study designs, including both case-control studies and cohort studies [13-19,21]. These studies, which have primarily focused on the vaccine's effectiveness in preventing invasive S. pneumoniae infections (rather than pneumonia), have produced a wide range of estimates, from 0% to 86%, but at least some of this variation appears to have been due to differences in the level of immunosuppression present in the HIV-infected individuals included in the studies. In individuals with higher CD4 T cell counts and/or receiving effective anti-retroviral regimens, the estimates of the effectiveness of pneumococcal polysaccharide vaccine have typically been in the range of 40 to 80%. On the other hand, there is no evidence from previous studies that the vaccine is effective in severely immunocompromised HIV-infected individuals.

Because Brazil has been at the forefront of making effective anti-retroviral drug regimens available to HIV-infected individuals, irrespective of their ability to pay, the patient population from which our cases and controls came typically were receiving anti-retroviral drugs if indicated under Brazil's guidelines for the care of patients with HIV/AIDS. We found that being on anti-retroviral medications at the time of the last outpatient visit was associated with substantial protection against invasive pneumococcal infection.

## Conclusion

In this patient population, we were unable to demonstrate that pneumococcal polysaccharide vaccine was protective against invasive pneumococcal infection. While the vaccine is relatively inexpensive and safe, its effectiveness among HIV-infected adults is uncertain and the argument in favor of giving it routinely to such patients is not supported by consistent evidence of its effectiveness. If given to such patients, however, it should be given before advanced immunosuppression has developed, in order to maximize the likelihood that it will be effective.

## Competing interests

The author(s) declare that they have no competing interests.

## Authors' contributions

MASMV conceived of the study, participated in its design, coordination and statistical analysis and draft the manuscript.

ALR conceived of the study, participated in its design and draft the manuscript.

WE performed the statistical analysis and helped to draft the manuscript.

EAC conceived of the study, participated in its design and helped to draft the manuscript.

All authors read and approved the final manuscript.

## Pre-publication history

The pre-publication history for this paper can be accessed here:


